# Gut Leakage of Fungal‐Related Products: Turning Up the Heat for HIV Infection

**DOI:** 10.3389/fimmu.2021.656414

**Published:** 2021-04-12

**Authors:** Stéphane Isnard, John Lin, Simeng Bu, Brandon Fombuena, Léna Royston, Jean-Pierre Routy

**Affiliations:** ^1^ Infectious Diseases and Immunity in Global Health Program, Research Institute, McGill University Health Centre, Montreal, QC, Canada; ^2^ Chronic Viral Illness Service, McGill University Health Centre, Montreal, QC, Canada; ^3^ CIHR Canadian HIV Trials Network, Vancouver, BC, Canada; ^4^ Division of Hematology, McGill University Health Centre, Montreal, QC, Canada

**Keywords:** fungi, inflammation, HIV, beta-D-glucan [BDG], immune activation

## Abstract

The intestinal epithelial layer serves as a physical and functional barrier between the microbiota in the lumen and immunologically active submucosa. Th17 T-cell function protects the gut epithelium from aggression from microbes and their by-products. Loss of barrier function has been associated with enhanced translocation of microbial products which act as endotoxins, leading to local and systemic immune activation. Whereas the inflammatory role of LPS produced by Gram-negative bacteria has been extensively studied, the role of fungal products such as β-D-glucan remains only partially understood. As HIV infection is characterized by impaired gut Th17 function and increased gut permeability, we critically review mechanisms of immune activation related to fungal translocation in this viral infection. Additionally, we discuss markers of fungal translocation for diagnosis and monitoring of experimental treatment responses. Targeting gut barrier dysfunction and reducing fungal translocation are emerging strategies for the prevention and treatment of HIV-associated inflammation and may prove useful in other inflammatory chronic diseases.

## Introduction

Gut damage and increased gut permeability constitute hallmarks of both acute and chronic phases of HIV infection (1, 2). CD4+ T cells loss in the gut mucosa, including interleukin (IL)-17-producing T-helper cells (Th17), disturbs mucosal homeostasis and contributes to epithelial gut damage (3). HIV-associated loss of epithelial integrity induces the non-physiological passage of microbial by-products from the gut lumen into the systemic circulation, referred to as microbial translocation. Brenchley et al. first reported in 2006 that increased plasma levels of the Gram-negative bacterial cell wall antigen lipopolysaccharide (LPS) triggers systemic immune activation in both people living with HIV (PLWH) and SIV-infected rhesus macaques (2), and eventually contributes to disease progression in PLWH (4–8). Moreover, in macaque models, gut epithelium damage precedes immune activation (9). Although antiretroviral therapy (ART) successfully controls HIV replication and prevents AIDS, the gut epithelium is not fully repaired in long-term ART-treated PLWH (4, 10, 11). As such, microbial translocation persists along with systemic immune activation in ART-treated PLWH (4, 12–15). This chronic inflammation in ART-treated PLWH likewise increases the risks of non-AIDS comorbidities such as cardiovascular and metabolic diseases, neurocognitive dysfunction and cancer (16). Therefore, understanding the link between epithelial gut damage and systemic immune activation in PLWH is crucial in both ART-naïve and ART-treated PLWH.

On the luminal side of the gut epithelium lives a complex microbiota. Different in almost every individual, the gut microbiota composition is well-controlled by both the microbiota itself and the host. Composed of bacteria, fungi, archaea, protozoa and viruses, the microbiota plays key physiological and immune roles through the metabolism of different nutrients, regulation of the immune system and control of pathogen invasion. Yet, microbiota composition studies predominantly focus on bacteria. As such, microbial translocation of bacterial products such as LPS is primarily studied alongside the subsequent immune response, quantified by host factor soluble CD14 (sCD14) produced by macrophages/monocytes in response to LPS stimulation, and LPS-binding protein (LBP) mostly produced by the liver in the presence of LPS.

Fungal mass constitutes the second player after bacterial mass in the composition of gut microbiota. Fungi are thus found in the gut of all healthy individuals and PLWH (17, 18), with *Saccharomyces cerevisiae*, *Malassezia restricta* and *Candida albicans* being the most often found in stools. As such, one can hypothesize that fungal product would also translocate into the circulation in the presence of a leaky gut. (1→3)-β- D-Glucan BDG is a major cell wall component of most fungi and is used as a clinical biomarker for diagnosing and managing invasive fungal infection (IFI). Although other cell wall molecules such as mannans and galactomannans are also common across fungi species colonizing humans, BDG is the only marker associated with fungal translocation in PLWH. Morris et al. first showed elevated plasma levels of BDG in PLWH in 2012 (19). Since then, several other groups including ours reported an association between BDG and epithelial gut damage, immune activation, inflammation, and risk of developing non-AIDS comorbidities (4, 15, 20–24). These findings suggest a significant role for BDG in chronic immune activation and the development of non-AIDS comorbidities in PLWH, although the mechanisms involved remain poorly understood.

The development of non-AIDS comorbidities despite long-term ART represents the main concern in care for PLWH (16, 25–27). As fungal translocation appears to play a key role in immune activation, understanding mechanisms behind this phenomenon could help in designing novel therapies aiming at improving the quality of life of ART-treated individuals. Herein, we delve into the literature regarding the contribution and mechanism by which fungal translocation induces systemic immune activation and non-AIDS comorbidities in PLWH.

## Evidence of Gut Leakage of Fungal Products in Animal Models

Fungi are peaceful colonizers of the skin but also lungs and genital tract of most mammals including humans. They’re also naturally present in the gut microbiota in absence of invasive fungal infection (IFI) (28). However, fungal products that are found in the blood usually result either from IFI or from translocation of fungal products predominantly from the gut (17, 18, 29–31).

The gastrointestinal tract (GI) encompasses multifaceted physical and immunological barriers preventing translocation of microbes and their by-products, while allowing for the absorption of nutrients. The gut mucosa is protected by both physical and immune components: the mucus and epithelial tight junctions on the apical pole of intestinal cells form a physical barrier; patrolling leukocytes in the lamina propria constitute an immune barrier ensuring that any translocated pathogens are phagocytosed, cleared, and/or transferred to mesenteric lymph nodes.

Fungi in the gut microbiota are abundant in mammals and play key roles in the balance between bacteria and other communities, as well as immune development in mice (32, 33). Animal models of gut damage that are frequently used include oral treatment with Dextran sulfate sodium (DSS), which impairs the gut epithelium and creates an experimental colitis (34). Upon DSS treatment, fungal products were found in the systemic circulation in different mice models (35, 36). Moreover, translocated fungal products, including BDG, were shown to participate in inflammation (37).

These mouse models suggest that upon gut damage, microbial translocation of fungal products occurs and participates in inflammation induction. As fungi are also present in the gut microbiota of non-human primates, studies could be performed to confirm the origin of translocated fungal products in different pathologies (31, 38).

## Evidence of Gut Leakage of BDG in People Living With HIV

Increased gut permeability is a hallmark of HIV infection and has been shown to increase microbial translocation and inflammation (2). Markers of gut damage, Zonulin and intestinal fatty acid binding protein (I-FABP), as well as the marker of gut permeability regenerating islet-derived protein 3-α (REG3α) were found at higher levels in PLWH (10, 39). Beside translocation of bacterial products, higher circulating levels of fungal products were also found in PLWH, suggesting microbial translocation of fungal products (reviewed in **Table 1**).

**Table 1:** Main studies assessing the influence of fungal translocation in people living with HIV.

	Country	Sample size	Population	Study design	Main findings	Reference
2012	USA	132	CHI, mostly ART+	Cross sectional	Higher BDG values associated with inflammation, CD8 T-cell activation, and pulmonary abnormalities.	(19)
2015	USA	41	CHI ART+	Cross sectional	Blood BDG levels correlated with neopterin levels and tended to correlate with TNF-α levels.	(15)
2016	USA	11	Early infection, before and after ART	Cross sectional	Blood BDG and sCD14 levels were associated with lower colonization of *Lactobacilli* in stools.	(23)
2016	USA	21	CHI ART+	Cross sectional	Higher blood BDG levels were associated with neurocognitive dysfunction.	(22)
2018	USA	451	Before and after ART	Cross sectional	suPAR and BDG plasma levels after ART initiation were associated with increased risk of non-AIDS comorbidities.	(40)
2019	Canada	146	Early and chronic, ART naïve or ART+	Longitudinal Cross sectional	Plasma BDG levels were higher in chronically infected people than early infection, and were associated with inflammation and immune activation.	(4)
2019	USA	231	ART naïve before and after ART, comparison of TDF/FTC, ATV +DRV, or RAL	Longitudinal	BDG increased after ART initiation, in association with increase in body fat.	(20)
2019	USA	61	CHI ART+	Cross sectional	BDG levels in plasma were associated with neurocognitive function.	(21)
2019	USA	176	CHI ART+ and uninfected controls	Cross sectional	Lower levels of BDG in HIV+ participants compared to uninfected controls. BDG levels correlated with levels of inflammation markers in HIV+ participants. No difference in levels of anti-fungal antibodies were found.	(24)
2020	USA	14	CHI ART+, compared to people with liver cirrhosis and healthy controls	Longitudinal and cross sectional	Oral challenge with BDG rich food did not increase blood levels of BDG.	(41)
2020	Uganda	171	Children (2-10 years old) HIV+ ART+, and uninfected, HIV exposed or not	Cross sectional	Blood BDG levels were higher in HIV infected children. In children with a history of breastfeeding, BDG levels correlated with soluble TNF receptor levels.	(42)
2020	Uganda	101	Children (10-18 years old) HIV+ ART+, and uninfected, HIV exposed or not	Cross sectional	Blood BDG levels were higher in HIV infected children. BDG levels were associated with immune activation in monocytes and T-cells.	(43)
2020	Canada	11	CHI ART+	Longitudinal	24 hours follow-up of participant showed no significant variations of BDG levels in blood.	(44)
2021	The Netherlands	40	CHI ART^+^ and uninfected controls	Cross-sectional	A higher proportion of ART-treated PLWH had detectable BDG levels in blood, and those levels were associated with inflammatory markers.	(14)
2021	Canada	145	CHI ART+ and uninfected control	Cross-sectional	BDG levels were associated with subclinical coronary atherosclerosis plaque in PLWH but not uninfected controls.	(45)

CHI, chronic HIV infection; ART, antiretroviral therapy; TDF, tenofovir disoproxil fumarate; FTC, emtricitabine; ATV, atazanavir; DRV, darunavir; RAL, raltegravir.

Morris et al. were the first to report elevated levels of fungal product BDG in the blood of PLWH, grouping together ART-treated and viremic untreated individuals (19). Clinically, higher circulating BDG levels were associated with absence of ART, higher viral load and lower CD4 T-cell count (19).

Weiner et al. found lower levels of BDG in PLWH compared to uninfected controls, although high levels of BDG were also found in the control group (24). Interestingly, this study also showed no difference in levels of anti-*Saccharomyces* antibodies (ASCA) between both groups (IgG and IgA).

We previously compared plasma levels of distinct gut damage and microbial translocation markers in different groups of PLWH without IFI and showed that plasma levels of BDG were higher in PLWH compared to uninfected controls, while galactomannan levels were low and similar between both groups. We also found higher levels of BDG in chronically infected PLWH compared to those in the early phase of the infection (4). Surprisingly, BDG levels were not statistically lower in ART-treated PLWH compared to their ART-naïve counterpart. Moreover, those levels correlated with markers of gut damage I-FABP and gut permeability REG3α in PLWH and uninfected controls, in accordance with the hypothesis that fungal translocation originates from gut microbiota (4, 10). Also, BDG and LPS levels correlated, and both were hypothesized to originate from the gut. Moreover, after a 2-year follow-up, PLWH not taking ART had increased levels of blood BDG levels, while those treated during the early phase of the infection had stable BDG levels (4). Early ART initiation was also associated with lower BDG levels, suggesting that early ART decreases the magnitude of gut damage and prevents further BDG translocation. All in all, these results demonstrated that fungal BDG translocation occurs in PLWH and suggest that these molecules originate from the gut.

## Validating BDG as a Marker of Microbial Translocation in PLWH

Recent findings tend to validate BDG as a marker of microbial translocation. Indeed, BDG can be found in several types of food including oatmeal, mushrooms, and seaweed. One would expect that increased intake of food rich in BDG might lead to its increased absorption. Therefore, Hoenigl et al. designed a clinical trial where people were fed with high-BDG food in a controlled environment (41). This study included participants with advanced HCV-associated liver cirrhosis as positive controls, as those patients have elevated microbial translocation levels (46–49). Other included participants constituted of PLWH with detectable viral loads, ART-suppressed PLWH, and HCV negative/HIV negative controls. Although BDG testing of the BDG-rich food confirmed an elevated concentration, no significant variation of plasma BDG levels were detected in any participants up to 8 hours after food intake. This study strengthened the hypothesis that translocated BDG is originating from fungal communities in the GI tract rather than from food intake.

In addition, we also demonstrated that BDG levels were stable throughout 24 hours in ART-treated PLWH, as opposed to LPS levels (44). Interestingly, LPS levels increased after lunch and dinner, and decreased during the night, while BDG levels were stable over 24 hours. Although we were not able to exclude a circadian regulation mechanism, we hypothesized that detoxification of LPS might explain its variation. Indeed, BDG levels were stable upon ART initiation in PLWH, when gut damage marker levels decreased, suggesting that translocated BDG is not detoxified as efficiently as LPS (11, 50).

## Consequences of BDG Translocation in PLWH

### Inflammation

Translocated products are recognized by the immune system as pathogen-associated molecular patterns (PAMPs) and induce inflammation. As such, several studies found associations between BDG and inflammation or immune-activation markers in PLWH.

Morris et al. found that participants with higher BDG levels had increased circulating levels of inflammatory cytokines IL-8 and tumor necrosis factor α (TNF-α), as well as higher levels of activated CD8 T-cells in ART-naïve and ART-treated PLWH (19).

Interestingly, in PLWH in the primary phase of the infection starting ART, circulating BDG, but not LPS, levels were inversely associated with gut colonization of *Lactobacilli*, which are associated with reduced colon inflammation (23). This association was demonstrated 12 weeks after ART initiation and tended to persist 12 weeks later.

In ART-treated PLWH, Hoenigl et al. found that BDG levels, although in the normal range (below 60 pg/mL), were associated with plasma levels of Neopterin, a marker of inflammation, and tended to correlate with plasma levels of pro-inflammatory cytokines IL-6 and IL-8 (15). However, no association between BDG levels and the marker of bacterial-related inflammation sCD14 could be observed.

Higher BDG levels have been associated with markers of disease progression: in ART-naïve PLWH, we found an association between viral load and BDG, but not LPS levels (4). Furthermore, in both ART-naïve and ART-treated PLWH, BDG levels were associated with lower CD4 count and lower CD4/CD8 ratio, indicating a link between BDG translocation and markers of disease progression.

BDG levels were also associated with pro-inflammatory cytokines IL-6, IL-8 and CXCL13 in blood, as well as the frequency of activated blood CD4 and CD8 cells (4, 51) (**Table 1**). Moreover, Weiner et al. showed that levels of BDG, but not ASCA, in PLWH correlated with inflammation markers such as IP-10, IL-6, markers of monocyte/macrophage activation sCD14 and sCD163 and percentage of activated CD4 and CD8 T-cells (24).

In Ugandan ART-treated children, BDG levels were also elevated compared to HIV-exposed or unexposed children (42, 43). Also, BDG levels were associated with levels of the soluble TNF-receptor, another marker of inflammation (42).

Van der Heijden reported that PLWH with higher levels of BDG exhibited higher plasma levels of the inflammatory marker IL-1β, as well as higher response of monocytes to imiquimod or *Mycobacterium tuberculosis* stimulations (14).

Altogether, these findings indicate that fungal translocation of BDG is associated with inflammation, in both ART-naïve and ART-treated individuals, possibly participating in disease progression.

### Non-AIDS Comorbidities

Persisting inflammation, even in ART-treated PLWH, is associated with increased risk of developing non-AIDS comorbidities including cardiovascular and metabolic diseases, and neurocognitive dysfunction. As translocation of fungal products has been associated with inflammation, the link between BDG and those comorbidities was investigated in several studies.

In 2018, Hoenigl et al. performed a cross sectional analysis of 451 PLWH, followed up to 11 years after ART initiation, and looked at the frequency of non-AIDS comorbidities, including myocardial infarction or stroke, non-AIDS malignancy or serious bacterial infection, or death from a non-AIDS related event. Among other markers of inflammations, only blood levels of soluble urokinase plasminogen activator receptor (suPAR), a marker of T-cell and monocyte activation, as well as BDG, were associated with non-AIDS comorbidity occurrence (40). Interestingly, only post-ART and pre-comorbidity BDG levels were associated with development of those comorbidities, independently of CD4 count but not smoking status pre-event.

Morris et al. found that PLWH with higher BDG levels had higher frequency of cardiopulmonary abnormalities including reduced diffusing capacity for carbon monoxide, higher pulmonary artery systolic pressure and increased tricuspid regurgitant jet velocity (19).

We also showed an association between plasma BDG levels and subclinical coronary atherosclerosis plaque in ART-treated PLWH but not uninfected controls, independently of age sex and other typical factors. Interestingly, we found that BDG levels were more strongly associated with plaque prevalence than age, smoking habits, hypertension, statin use or obesity (45).

Moreover, a study assessing metabolic and weight changes showed that after ART-initiation, blood BDG levels increased two years after ART initiation, and were associated with larger trunk and total body fat accumulation (20).

Several studies have shown a link between BDG levels and cognitive functions in PLWH. Plasma BDG levels were associated with Global Deficit Score in ART-treated PLWH (22). Interestingly, this study showed that the 2 participants (out of 21) who had the worst deficit were also the only ones with elevated BDG levels in cerebrospinal fluid. Also, although IL-8 levels in plasma were associated with the deficit score, no correlation between BDG levels and IL-8 levels was observed in this study (22). The same team expanded such findings in 61 ART-treated PLWH and found that suPAR and BDG plasmatic levels were associated with the Global Deficit Score, independently of CD4 T-cell count (21) **(** **Table 1** **).**

Although BDG appears as a new marker of non-AIDS comorbidities, current observations rely on associations only. More studies are thus needed to puzzle out the mechanism linking fungal translocation and comorbidities.

## Detection of Fungal Products in PLWH—Insights on Mechanisms

Fungal PAMPs induce inflammation following their detection by pattern recognition receptor (PRRs) expressed on different cell types. Fungal PRRs include C-type lectin receptors such as Dectin-1, Toll-like receptor 2, integrins, scavenger receptors, and hyaluronic acid receptors (52). The receptor ephrin type-A receptor 2 (EphA2) has also been shown to mediate detection of fungal BDG in the mouth and upper GI, inducing protective innate immunity (53). EphA2 is also expressed at lower levels throughout the gut. Whether this receptor is implicated in fungal product induction of inflammation in PLWH has not been elucidated yet.

### Effect on Antigen Presenting Cells and Neutrophils

Antigen presenting cells (APCs) are specialized in the detection of pathogens through conserved PAMPs, allowing the development of appropriate immune responses. APC include dendritic cells, macrophages/monocytes, and B cells, and are highly abundant in tissue, notably in the gut.

**Figure 1:**
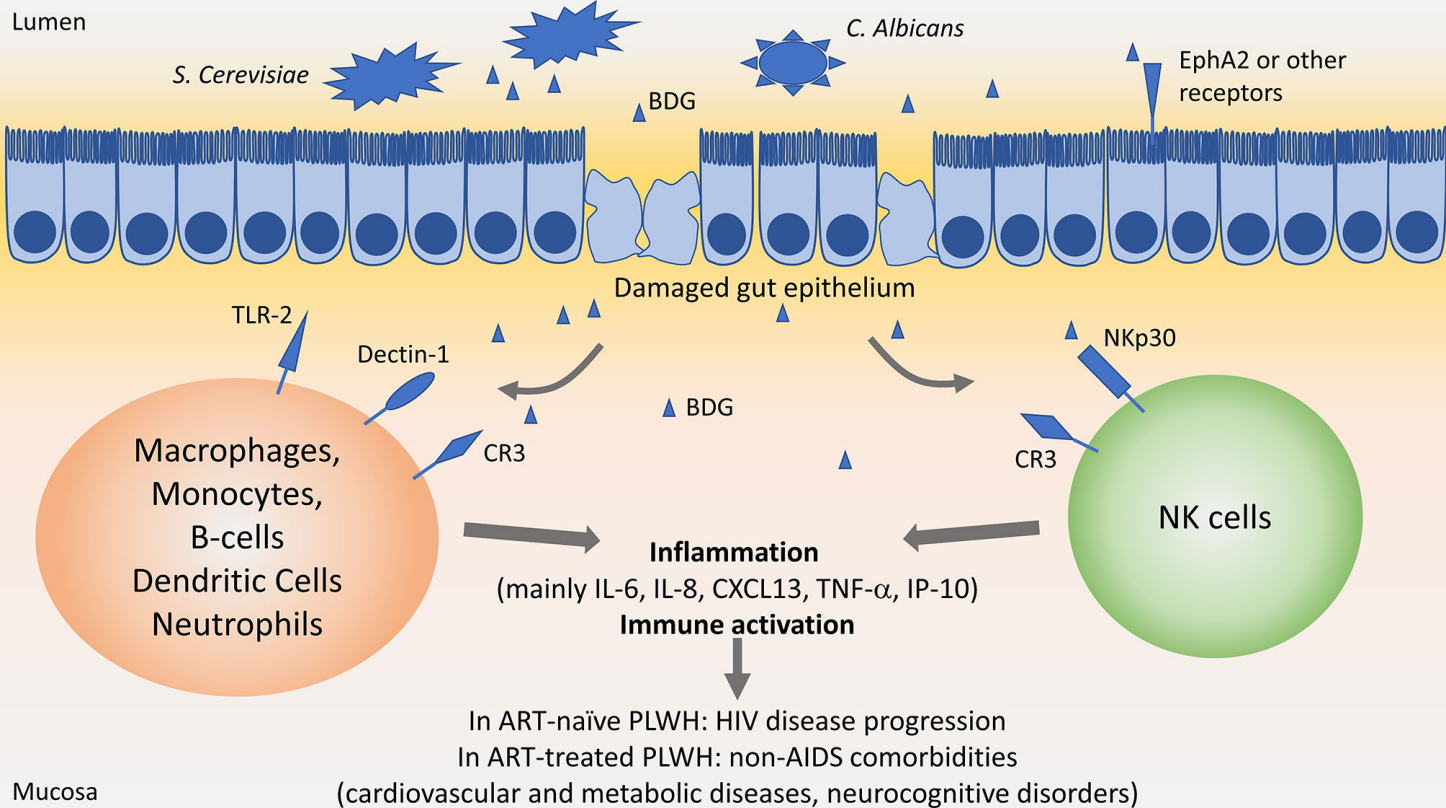
Influence of β-D-Glucan in people living with HIV. In the gut lumen, *Saccharomyces Cerevisiae* and *Candida albicans* are largely present in the microbiota. Upon HIV-associated epithelial gut damage, fungal products such as β-D-Glucan (BDG) translocate in the mucosa. BDG is recognized by immune cells through Toll-like receptor 2 (TLR-2), Dectin-1, complement receptor 3 (CR3) or NKp30, activating immune cells and inducing inflammation. Persisting inflammation has been associated with disease progression in people living with HIV (PLWH) not taking antiretroviral therapy (ART), and with increased risk of non-AIDS comorbidities in ART-treated PLWH.

APCs can sense fungi through different receptors including Dectin-1, Toll-like receptor 2 (TLR2) and Complement receptor 3 (CR3) (**Figure 1**).

Dectin-1 is the main receptor interacting with BDG on macrophages, monocytes, dendritic cells, B-cells, and neutrophils (54–57). Expressed at the cell surface, Dectin-1 recognizes circulating or membrane-bound BDG, activating the NF-κB pathway through activation of the CARD9/BCL10/MALT1 complex.

TLR2 is expressed on Dendritic cells, macrophages, and monocytes, and also activates the NF-κB pathway through activation of MyD88 upon recognition of soluble or particulate BDG.

Both stimulation of Dectin-1 and TLR2 on macrophages and monocyte induce the secretion of pro-inflammatory cytokines such as IL-6, IL-8, TNF-α, as well as anti-inflammatory mediator IL-10 (55) (**Figure 1**). It is worth noting that stimulation of monocytes with BDG induced internalization of Dectin-1 and decreased its surface expression as soon as 30 min after stimulation (54, 55). Size of BDG molecules play a key role in the induction of inflammatory responses, with larger-sized BDG inducing higher IL-1β, IL-6 and IL-23 secretion compared to smaller-sized BDG. However, secretion of chemokines involved in recruitment and maturation of T-cells was not affected by BDG size (58).

CR3 can also trigger BDG recognition on macrophages, monocytes, and neutrophils. However, neutrophils recognize BDG through CR3 only after opsonization with complement (59).

*In vitro* or animal models indicated that APC and neutrophils secrete inflammatory cytokines when stimulated by BDG, however the indication of such direct effect in PLWH is still lacking. As an initial foray, we found that circulating levels of BDG, but not LPS, inversely correlated with Dectin-1 expression on monocytes in PLWH (4), suggesting a direct interaction between BDG and its receptor Dectin-1 on monocytes. To validate this mechanism, we stimulated PBMC *in vitro* with *Saccharomyces Cerevisiae*-extracted BDG and found decreased Dectin-1 expression on monocytes at 24 and 48 hours. LPS did not induce such variation [personal communication (60)]. Moreover, stimulation of monocytes and macrophages with BDG was shown to primarily induce IL-1β and IL-8, which correlated with BDG levels in plasma samples in PLWH.

### Detection by NK Cells

NK cells are a key player of innate immunity responsible for eliminating infected cells, cancer cells, as well as fungi. The main fungal receptor on NK cells is NKp30, also called Natural cytotoxicity triggering receptor 3 (NCR3). Recent work has shown that NKp30 recognizes membrane bound BDG, allowing elimination of fungal cells. NKp30 is required for elimination of *Cryptococcus* in a mouse model (61, 62). As such, NKp30 is the PRR responsible for direct recognition of fungus and BDG by NK cells. Unexpectedly, soluble BDG also binds to NKp30, activating NK cells and allowing the secretion of cytotoxic molecules Perforins and Granzymes (61, 62). Addition of BDG to NK increased *Candida*-killing activity. Earlier, this group showed that NKp30 surface expression is reduced on NK cells from ART-treated PLWH (62). We later confirmed those results in both ART-naïve and ART-treated chronically infected PLWH and also found that surface NKp30 expression was negatively correlated with circulating BDG but not LPS levels (4). *In vitro*, stimulation with *S. Cerevisiae-*extracted BDG but not with LPS decreased NKp30 expression at 24 and 48h (60), confirming the direct role of BDG in reducing NKp30 expression. Reduced NKp30 expression was associated with lower cytotoxic function against fungi and cancer cells (61–63).

Altogether, these findings indicate that BDG has a direct stimulating role of NK cells, including in PLWH on ART. This could lead to inflammation and decreased efficiency in infection or cancer suppression, leading to increased non-AIDS comorbidities.

### BDG and Trained Immunity in HIV

Recent findings have put fungal products under the spotlight as they robustly induce trained immunity. This type of innate immune memory has been shown to be induced by β-glucans (including BDG) and BCG vaccines (64, 65). Trained immunity is defined as the process by which a stimulation programs a cell to respond with greater efficiency to a second stimulation after returning to steady state following the first stimulation. Trained immunity is functionally different from priming and differentiation and opposed to tolerance (64). In animal and human models, BDG has been shown to activate immune cells, especially monocytes, and induce epigenetic changes allowing those cells to respond with greater intensity to a second stimulation. Trained immunity is not antigen restricted as it potentiates the response to subsequent stimuli differently from the first antigen encounter and has been shown to act throughout the body *via* modulation of hematopoiesis and cell trafficking.

Whether translocated BDG plays a role in inducing trained immunity in PLWH is still unknown. In 2020, Van Der Heijden identified a link between circulating BDG levels and a trained immunity phenotype in ART-treated PLWH (14). Whether this phenotype is induced by trained immunity or priming of monocytes will have to be elucidated in further studies.

However, several indications lead to the hypothesis that this trained immunity is unlikely in PLWH. The first clue concerns the dynamics: most models of trained immunity require the removal of the initial stimulus to potentiate a second response, while BDG persists chronically even at low levels in PLWH. The second clue relies on the complexity of microbial translocation in PLWH: BDG translocation is accompanied by other microbial products such as LPS, inducing various inflammatory signals, while trained immunity has been shown to mostly rely on single instances of antigenic stimulation with β-glucans or BCG. The last hint is clinically relevant: glucan-induced trained immunity has been shown to increase protective responses to diverse infections and cancer, while PLWH have increased risks of both infection and cancer. However, trained immunity could also participate in sustained chronic inflammation such as in atherosclerosis, notably through the recognition of oxidized low-density lipoprotein particles (66). Indeed, BDG levels have been linked with cardiovascular disease in PLWH (19, 40, 45).

Hence, and due to the difficulty in deciphering priming from trained immunity, the influence of microbial translocation of BDG on trained immunity in PLWH should be assessed in future studies.

## Targeting Fungal Translocation in PLWH

We and others have shown that starting ART as early as possible appears to stabilize BDG levels, in accordance with current guidelines recommending ART initiation as soon as the diagnostic is confirmed (4, 20). Therefore, as fungal translocation has been associated with inflammation and non-AIDS comorbidities in ART-treated PLWH, strategies targeting fungal translocation are needed.

Treatment with the antifungal agent fluconazole in ART-treated PLWH with neurocognitive disorders barely changed levels of markers of inflammation IL-1α, IL-6, IL-8 and IP-10 (67). However, levels of fungal products translocation have not been assessed in this study, rendering it difficult to draw conclusions on the effect of anti-fungal treatment on fungal microbial translocation.

Specific strategies have not been developed to prevent microbial translocation of fungal products in ART-treated PLWH. Fecal microbiota transplantation (FMT) could influence the mass of the mycobiome and allow improvement of gut epithelium integrity, reducing fungal translocation (68). Although several pilot trials of FMT have been initiated in PLWH, few have studied fungal translocation before and after treatment. In 2020, a study by Utay et al. consisting in six weekly FMT rounds in six ART-treated PLWH reported neither significant variations of circulating BDG levels, nor changes in inflammation and gut permeability markers I-FABP (69).

However, BDG levels have been used as markers of translocation in several other clinical trials:

In one study, metformin was expected to decrease inflammation in ART-treated PLWH (70, 71). Pilot results showed that 3 months of metformin treatment in addition to ART slightly decreased the marker of inflammation sCD14, but did not decrease LPS nor BDG translocation (72).

In a randomized placebo-controlled double-blind study, dipyridamole treatment was shown to increase extracellular adenosine levels and decrease CD8 T-cell activation in ART-treated PLWH (73). However, this treatment did not modify BDG levels in either group (74).

## Conclusion

Translocation of fungal products, mainly inferred from BDG levels in the blood, has been shown to be associated with inflammation and comorbidities in PLWH. Whether BDG contributes directly to inflammation remains unknown, although assessment of BDG-receptors in PLWH pledges in this favor. Further studies are required to examine the role of fungal translocation in PLWH, especially those receiving ART. Overall, BDG appears as a robust biomarker of microbial translocation linked with inflammation and non-AIDS comorbidities in PLWH. Targeted strategies are critically needed to reduce the contribution of fungal translation to inflammation in PLWH, and eventually improve the quality of life of this population.

## Author Contributions

SI wrote the first draft, constructed the figure and table, and made revisions to the final draft of the manuscript. JL, SB, BF, and LR participated in the discussion and critically read and edited the manuscript. J-PR designed the review and critically revised the manuscript. All authors contributed to the article and approved the submitted version.

## Funding

This work was funded by the Canadian Institutes of Health Research (CIHR; grants MOP 103230 and PTJ 166049), the Vaccines & Immunotherapy Core of the CIHR Canadian HIV Trials Network (CTN, grant CTN 257, CTN PT032, and CTNPT038), the CIHR-funded Canadian HIV Cure Enterprise (CanCURE) Team Grant HB2-164064 and réseau Fonds de la recherche-Santé (FRQ-S) SIDA Maladies infectieuses and thérapies cellulaires. SI is a post-doctoral fellow supported by the FRQ-S and CIHR-CTN. BF is supported by a William Turner award from the McGill University Health Centre. LR is a post-doctoral fellow supported by the “Fonds de perfectionnement” of the Geneva University Hospitals, Switzerland. J-PR is the holder of the Louis Lowenstein Chair in Hematology and Oncology, McGill University.

## Conflict of Interest

The authors declare that the research was conducted in the absence of any commercial or financial relationships that could be construed as a potential conflict of interest.
